# Expression and role of nestin in human cervical intraepithelial neoplasia and cervical cancer

**DOI:** 10.3892/ijo.2012.1473

**Published:** 2012-05-10

**Authors:** ATSUKI SATO, TOSHIYUKI ISHIWATA, YOKO MATSUDA, TETSUSHI YAMAMOTO, HIROBUMI ASAKURA, TOSHIYUKI TAKESHITA, ZENYA NAITO

**Affiliations:** 1 Departments of Pathology and Integrative Oncological Pathology, Nippon Medical School, Tokyo 113-8602;; 2 Division of Reproductive Medicine, Perinatology and Gynecologic Oncology, Graduate School of Medicine, Nippon Medical School, Tokyo 113-8603, Japan

**Keywords:** nestin, cervical intraepithelial neoplasia, uterine cervical cancer, immunohistochemistry, ME-180, cancer stem cell

## Abstract

Nestin expression reportedly correlates with aggressive growth, metastasis, poor prognosis and presence of cancer stem cells (CSCs) in various tumors. In this study, we determined the expression and role of nestin in cervical intraepithelial neoplasia (CIN) and cervical cancer. We performed immunohistochemical and *in situ* hybridization analyses of nestin in 26 cases for each stage of CIN and 55 cervical cancer tissue samples. To examine the role of nestin in cervical cancer cells, we stably transfected expression vectors containing nestin cDNA into ME-180 cells. We studied the effects of increased nestin expression on cell proliferation, cell motility, invasion as well as sphere and soft agar formation. Nestin was not localized in the squamous epithelium in normal cervical tissues, but it was weakly expressed in the basal squamous epithelium of CIN 1. In CIN 2, nestin was localized to the basal to lower 2/3 of the squamous epithelium, whereas in CIN 3, it was localized to the majority of the squamous epithelium. Nestin was detected in all cases of invasive cervical cancer. Nestin mRNA was expressed in both ME-180 and CaSki cells. Growth rate, cell motility and invasion ability of stably nestin-transfected ME-180 cells were not different from empty vector-transfected ME-180 (mock cells). However, the nestin-transfected ME-180 cells formed more colonies and spheres compared to the mock cells. These findings suggest that nestin plays important roles in carcinogenesis and tumor formation of cervical cancer cells. Nestin may closely correlate with regulation of CSCs.

## Introduction

Cervical cancer is the second most prevalent cancer in women worldwide and its occurrence has steadily increased among young women (1,2). Nearly half million new cases and 274,000 deaths occur each year due to this disease (3). More than 99% of cervical carcinomas are associated with human papilloma-virus (HPV), yet infection of HPV alone is not sufficient to cause cervical cancer (4).

Nestin is a protein belonging to the class VI intermediate filaments (IFs). It was first described as a marker of neuroectodermal stem and progenitor cells because it is expressed in proliferating cells during embryonic development of the central nervous system (CNS) (5). Nestin is a large protein (>1,600 amino acids), structurally similar to other IFs, with a highly conserved α-helical core domain of 300–330 amino acids flanked by amino- and carboxy-terminal domains (5,6). Nestin contains a short N-terminus and an unusually long C-terminus. It is assembled into IF by forming heterodimers with vimentin and desmin, but nestin cannot form homopolymers (7–10).

Nestin is also expressed in immature or progenitor cells in non-neuronal tissues (11–14). Strong nestin expression has been detected in oligodendroglial lineage cells, ependymocytes, Sertoli cells, enteroglia, hair follicle cells, podocytes of renal glomeruli, stellate cells, pericytes, islets, optic nerve and odontoblasts (15–21). Various tumors show increased nestin expression, including central nervous system (CNS) tumors, melanomas, gastrointestinal stromal tumors (GIST), prostate cancer, breast cancer and pancreatic cancer (22–27). Nestin has been detected in human gliomas, with expression more frequent in high-grade gliomas than in low-grade gliomas such as pilocytic astrocytomas (28,29). In malignant melanoma, stronger nestin expression is observed in advanced stage disease and in metastatic foci of melanoma cells (24,30). Nestin protein is most abundant at the infiltrating front of the tumors, suggesting that it plays important roles in melanoma cell migration and invasion (31). In breast cancer, nestin expression is associated with shorter survival and is an independent prognostic factor (32). Approximately 30% of pancreatic ductal adenocarcinoma (PDAC) cases show nestin immunoreactivity; nestin expression correlates with perineural invasion and the presence of cancer cells at the tumor resection margins in PDAC (6). Our group recently reported that expression levels of nestin directly correlate with migration, invasion and metastasis of pancreatic cancer cells (33).

Nestin has also received attention as a cancer stem cell (CSC) marker in various tumor cells including CNS tumors, uterine and cervical cancer, prostate, bladder, head and neck, ovarian, testicular and pancreatic cancers as well as malignant rhabdoid tumors (34–41).

However, the expression and role of nestin in cervical intraepithelial neoplasia (CIN) and cervical cancer are poorly understood. Here we report the correlation of nestin expression pattern with CIN progression. We found that nestin was expressed in all cervical cancer specimens. Increased nestin expression in cervical cancer cell lines stimulated colony and sphere formation *in vitro*.

## Materials and methods

### Non-cancerous, CIN and cervical cancer tissues

Cervical cancer samples (55 patients; mean age 50.4 years; median age 50 years; range 29–72 years) were obtained from patients undergoing surgery for invasive cervical cancer at the Department of Obstetrics and Gynecology of Nippon Medical School Hospital (Tokyo, Japan) from 2004–2010 (Table I). Twenty-six samples each for CIN 1–3 were obtained from cervical biopsies at the same hospital. For control samples, cervical biopsies from patients with chronic cervicitis (12 patients) were obtained. This study was carried out following the ethical guidelines of Nippon Medical School and the principles embodied in the Declaration of Helsinki, 2008.

### Immunohistochemistry

Paraffin-embedded sections (3 *μ*m) were subjected to immunostaining using a Histofine Simple Stain MAX PO (M) kit (Nichirei Corporation, Tokyo, Japan) for nestin as previously reported (42). Tissue sections were incubated with the anti-nestin antibody (1:200 dilution; R&D Systems, Westerville, OH) in phosphate-buffered saline (PBS) containing 1% bovine serum albumin (BSA) for 16 h. Bound antibodies were detected with the Simple Stain MAX PO (M) reagent, using diaminobenzidine tetrahydrochloride as the substrate. Negative control tissue sections were prepared by omitting the primary antibody.

### In situ hybridization analysis

A 235-bp BamHI-EcoRI cDNA fragment, corresponding to nucleotides 1045–1227 of the human nestin cDNA sequence, was subcloned into the pGEM-T vector and the result was confirmed by sequencing. Probes were labeled with digoxigenin-UTP using SP6 or T7 RNA polymerase and the DIG RNA-labeling kit (Roche Diagnostics GmbH, Mannheim, Germany). *In situ* hybridization was carried out as previously reported (43).

### Human cervical cancer cell line

ME-180 cells were obtained from the Cell Resource Center for Biomedical Research, Institute of Development, Aging and Cancer (Tohoku University, Sendai, Japan). CaSki cells were obtained from RIKEN BioResource Center (Ibaraki, Japan). ME-180 cells were grown in RPMI-1640 medium containing 10% heat-inactivated fetal bovine serum (FBS) at 37°C under a humidified 5% CO_2_ atmosphere.

### Construction of nestin expression vector

The full-length nestin cDNA fragment was ligated to the 3′-end of the human cytomegalovirus early promoter/enhancer in pAcGFP1-N1, a eukaryotic expression vector (Clontech Laboratories, Mountain View, CA). The correct orientation of the insert was verified by DNA sequencing. Nestin expressing and empty vectors were transfected using FuGENE HD transfection reagent (Roche Diagnostics GmbH). For stably-transfected cells, the cells were passaged and cultured with 1,000 *μ*g/ml of Geneticin.

### Quantitative real-time PCR of nestin in cervical cancer cell lines

Cervical cancer cells were grown in RPMI-1640 medium with 10% FBS for 48 h. Total-RNA extraction from cervical cancer cells was performed using a NucleoSpinII RNA kit (Takara Bio, Inc., Shiga, Japan). Then, cDNA synthesis was performed using High Capacity cDNA Reverse Transcription kit following the manufacturer’s protocol (Applied Biosystems, Carlsbad, CA). Real-time PCR for nestin and 18S rRNA was performed using the StepOnePlus real-time PCR system (Applied Biosystems) with specific primers for nestin (Hs00707120_s1) and 18S rRNA (Hs03928990_g1) and a TaqMan probe (7).

### Immunofluorescence staining and confocal laser microscopy

The same anti-nestin antibody used for the immunohistochemistry was also used for immunofluorescence staining of ME-180 cells. The cells were incubated with the anti-nestin antibody (1:50) in PBS containing 1% BSA for 18 h at 4°C. The cells were washed with PBS and then incubated with Alexa 568-conjugated goat anti-mouse IgG (Invitrogen Life Technologies, Carlsbad, CA) for 1 h. Then, cells were mounted with Vectashield mounting medium containing DAPI (Vector Laboratories, Inc., Burlingame, CA). Fluorescent images were acquired using a confocal laser scanning microscope Digital Eclipse TE 2000-E (Nikon Instech Co., Ltd., Tokyo, Japan) and were analyzed using the confocal microscope Digital Eclipse C1 control software EZ-C1 (version 2.30) (Nikon Instech Co., Ltd.).

### Morphological analysis of nestin-transfected ME-180 cells

Stably nestin-transfected ME-180 cells, empty-vector-transfected (mock cells) and nontreated cells (wild) were incubated in RPMI-1640 medium with 10% FBS for 48 h. Cell morphology was observed using a phase-contrast microscope (Nikon Eclipse TE2000-U).

### Anchorage-dependent growth assays

To examine the growth rates of nestin-transfected clones, a nonradioactive cell proliferation assay was performed. The cells were cultured in RPMI-1640 medium with 10% FBS at a density of 5×10^3^ in 96-well plates followed by incubation at 37°C in a humidified 5% CO_2_ atmosphere for 24, 48, 72, 96, 120 and 144 h. Then, the cells were incubated with WST-8 cell counting reagent (Wako Pure Chemical Industries, Ltd., Osaka, Japan) for 4 h at 37°C and the optical density of the culture solution in the plate was measured using an ELISA plate reader (Bio-Rad, Hercules, CA) at 450 nm. Alternatively, cells were plated at a density of 5×10^4^ cells/well and the cell number of each well was counted after 24, 48, 72 and 96 h using C-reader (Digital Bio Technology Co., Ltd., Kyungki-do, Korea).

### Growth of nestin-transfected ME-180 cells in soft agar

*In vitro* tumorigenicity was determined on the basis of cell growth in a soft agar colony assay. The flasks were covered with 2.5 ml RPMI-1640/0.5% agar/10% FBS. The upper layer consisted of 2 ml RPMI-1640/0.03% agar/10% FBS. 1×10^3^ and 2×10^3^ cells/well were seeded and incubated under a humidified 5% CO_2_ atmosphere at 37°C for 60 days. Images were obtained using Coolpix 5000 (Nikon Instech Co., Ltd.).

### Single-cell movement assay

ME-180 cells (8,000/well) were seeded onto a 4-well glass bottom dish and grown for 48 h. Cell movement was then monitored for 24 h using a Digital Eclipse TE 2000-E motorized inverted microscope taking pictures every 5 min. The total distance of individual cells covered within 24 h was determined using the Metamorph software 7.6 (Universal Imaging Corporation Ltd., Buckinghamshire, UK).

### Cell invasion assays

To assess the effect of nestin on cervical cancer cell invasion, an *in vitro* invasion assay using a modified Boyden chamber technique was carried out. Matrigel-coated inserts (8 *μ*m pore size and 6 mm in diameter; Becton-Dickinson and Co., Franklin Lakes, NJ) were used to assay cell invasion following the manufacturer’s instructions. Briefly, cell were suspended in 500 *μ*l serum-free medium and placed onto the upper component of the inserts at a density of 1×10^5^ cells. The lower compartment was filled with 750 *μ*l medium containing 10% FBS and the cells were incubated at 37°C in a humidified 5% CO_2_ atmosphere. After 48 h, the cells on the upper surface of the filter were fixed and stained with a Diff-Quick staining kit and counted under a light microscope. The cell number on each membrane was counted in 5 high-power fields (magnification, ×200).

### Sphere formation assay

To determine whether cervical cancer cells had CSC-like characteristics, we performed sphere formation assays. ME-180 cells (1,000/well) were plated in ultra-low attachment surface 24-well plate with serum-free medium supplemented with bFGF (10 ng/ml; ReproCell, Inc., Kanagawa, Japan) and EGF (20 ng/ml; R&D Systems). After 7 days, the number of spheres in each well was counted by phase-contrast microscope (Eclipse TE2000-U).

### Statistical analysis

All quantitative data are presented as mean ± standard error (SE) and assessed using Student’s t-test and Bonferroni/Dunn test. Computations were performed using the Stat View J version 5.0 software package (SAS Institute, Inc., Cary, NC).

## Results

### Immunohistochemical analysis of nestin in non-cancerous cervical tissue

**Figure 1:**
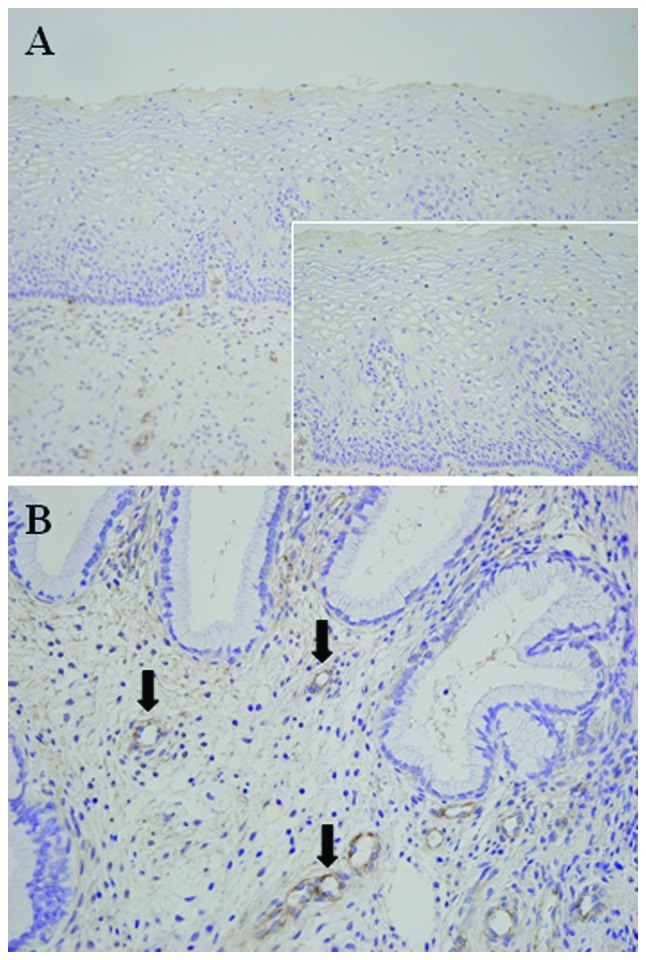
Immunohistochemical analysis of nestin in non-cancerous cervical tissues. (A and inset) Nestin protein was not localized in squamous epithelial cells, (B) but was localized in vascular endothelial cells. Original magnification, (A) ×200, inset ×400, (B) ×400.

Immunohistochemical analyses were performed to examine the localization of nestin protein in cervical tissues. In non-cancerous cervical tissue, nestin protein was not identified in squamous epithelial cells (Fig. 1A and inset). Nestin was not detected in cervical glands, whereas it was strongly expressed in stromal vascular endothelial cells (Fig. 1B).

### Immunohistochemical and ISH analyses of nestin in CIN tissues

**Figure 2:**
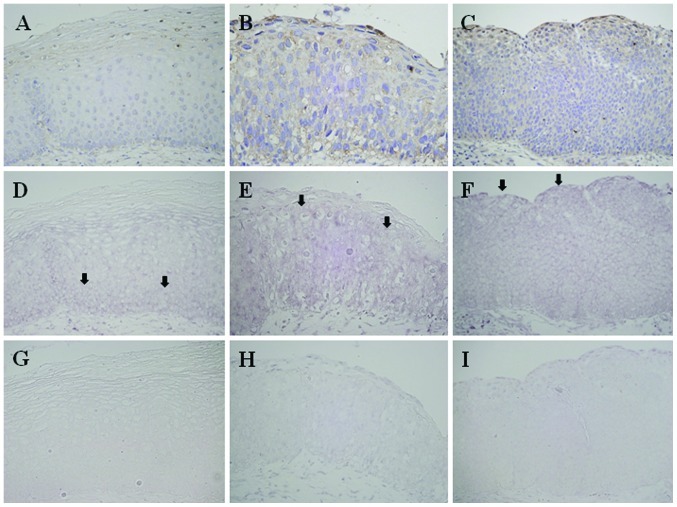
Immunohistochemical and ISH analyses of nestin in CIN. (A and D) Nestin protein and mRNA were localized at the basal to lower 1/3 of squamous epithelial layers in CIN 1, (B and E) between the basal layer and the lower 2/3 in CIN 2 and (C and F) in most squamous epithelial layers in CIN 3. (A–C) Immunohistochemistry; (D–F) *in situ* hybridization, antisense; (G–I) sense. Original magnification, (A, B, D, E, G and H) ×400 and (C, F and I) ×300.

In CIN 1, nestin protein and mRNA were weakly expressed or absent in the basal to lower 1/3 of squamous epithelial layers (Fig. 2A and D). In CIN 2, nestin protein and mRNA were localized between the basal layer and the lower 2/3 of squamous epithelial layers (Fig. 2B and E). In CIN 3, they were strongly expressed in most squamous epithelial layers (Fig. 2C and F). Sense probes did not show positive signals (Fig. 2G, H and I).

### Immunohistochemical and ISH analyses of nestin in cervical cancer tissues

**Figure 3:**
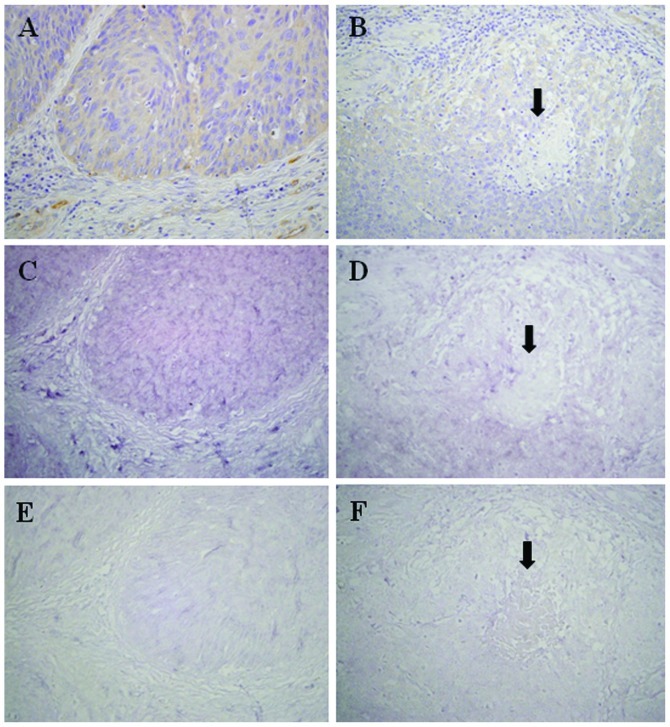
Immunohistochemical and ISH analyses of nestin in cervical cancer. (A, C and E) Nestin protein and mRNA were diffusely localized in cancer cell nests in the nonkeratinizing and (B, D and F) keratinizing tumor types. (A and B) Immunohistochemistry; (C and D) *in situ* hybridization, antisense; (E and F) sense. Arrows, keratinizing foci; original magnification, (A, C and E) ×200 and (B, D and F) ×400.

Nonkeratinizing cervical cancers showed solid and alveolar proliferative patterns, while keratinizing tumors showed foci of keratinization within nests of cancer cells (Fig. 3A and B). After mmunohistochemical analysis, nestin protein was detected in cancer cells of both the nonkeratinizing and keratinizing types in all examined cervical cancer samples. Nestin protein and mRNA were diffusely localized in cancer cell nests in the nonkeratinizing and keratinizing tumor cells (Fig. 3A–D). Nestin protein and mRNA were also expressed in endothelial cells of small-sized vessels near cancer cells. Nestin was not detected in the keratinizing areas within keratinizing types of cervical cancer (Fig. 3B and D). Sense probes did not show positive signals (Fig. 3E and F).

### Expression levels of nestin in cervical cancer cell lines

**Figure 4:**
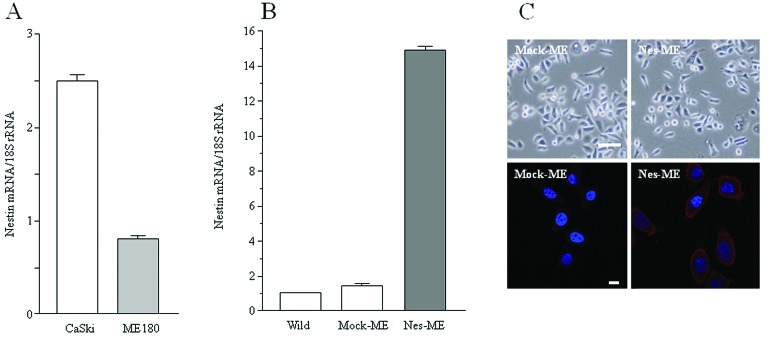
Cervical cancer cell lines and transfection of nestin expression vector into ME-180 cells. (A) Expression level of nestin mRNA was lower in ME-180 than in CaSki cells. Nestin expression vector was stably transfected into ME-180 cells. (B and C) Nestin mRNA and protein levels were higher in nestintransfected ME-180 cells (Nes-ME) than in control cells. (C) Phase-contrast images: bar, 1,000 *μ*m; immunofluorescence images: bar, 20 *μ*m.

To examine whether cervical cancer cells express nestin, Q-PCR was performed. Nestin mRNA was expressed in CaSki and ME-180 cervical cancer cell lines. Expression of nestin mRNA was 2.48-fold higher in CaSki cells than in ME-180 cells (Fig. 4A).

### Stably nestin-transfected ME-180 cells

To examine the roles of nestin in cervical cancer cells, we prepared nestinoverexpressing ME-180 cells. Full-length nestin cDNA was subcloned into pAcGFP1-N1 vector and stably transfected in the cells. The expression levels of nestin mRNA in mock cells (Mock-ME) and nestin-transfected ME-180 cells (Nes-ME) were examined by Q-PCR. Nestin mRNA levels were high in Nes-ME cells and low in Mock-ME and wild cells (Fig. 4B). Expression of nestin mRNA was 9.15-fold higher in Nes-ME than Mock-ME cells. Nes-ME cells did not show characteristic morphological alterations as compared with Mock-ME cells (Fig. 4C, upper panels). Immunofluorescent analysis showed that nestin protein is expressed in the cytoplasm of ME-180 cells, with stronger expression in Nes-ME than in Mock-ME cells (Fig. 4C, lower panels).

### Anchorage-dependent growth and cell motility assays

**Figure 5:**
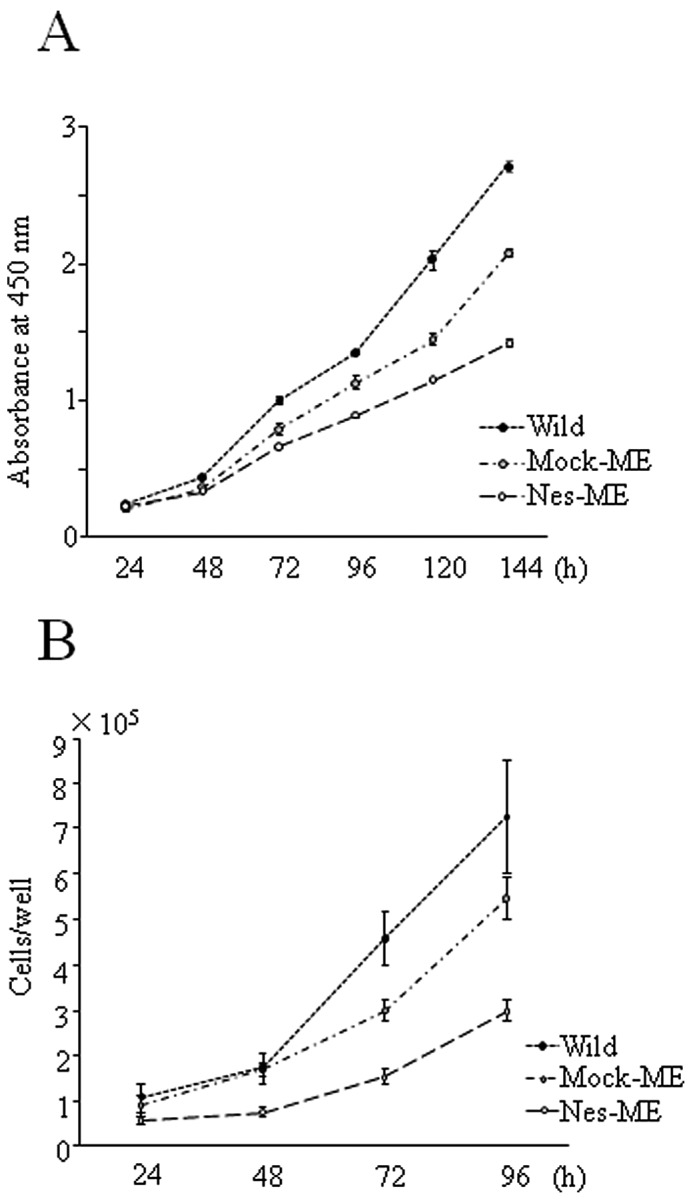
(A) Cell proliferation assay. The growth rates of Nes-ME cells using WST-8 reagent were lower than those of mock cells (Mock-ME), but this difference was not statistically significant. (B) Growth rates using cell count of Nes-ME cells were lower than those of Mock-ME cells, but the difference was not statistically significant.

**Figure 6:**
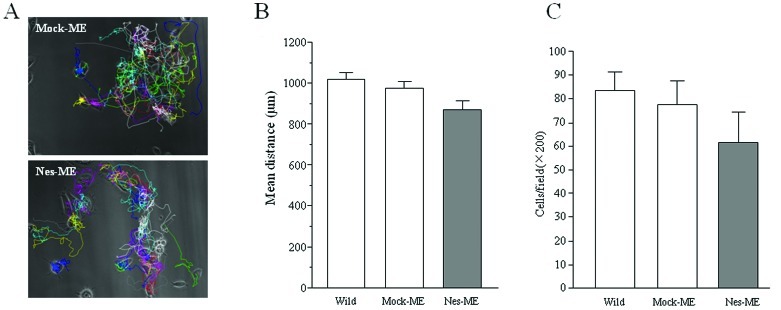
Cell motility assays. (A and B) The distances covered within 24 h by Mock-ME and Nes-ME cells showed no statistical difference. (C) In the invasion assay, Mock-ME and Nes-ME cells showed no difference in cell motility.

Nes-ME and Mock-ME cells were cultured for 24, 48, 72, 96, 120 and 144 h. Growth rates of stable Nes-ME cells were lower than those of Mock-ME cells, but this difference was not statistically significant (Fig. 5A). Growth rates using cell count of Nes-ME cells were lower than those of Mock-ME cells, similarly with the non-radioactive cell proliferation assay using WST-8 reagent (Fig. 5B). Next, a single-cell movement assay using time-lapse microscopy was performed to determine if nestin is involved in cellular motility. There was no significant difference between Nes-ME and Mock-ME cells( Fig. 6A and B). We also performed a modified Boyden chamber assay to assess cell invasion. There was no difference in invasive ability between Mock-ME and Nes-ME cells (Fig. 6C).

### Anchorage-independent growth assay

**Figure 7:**
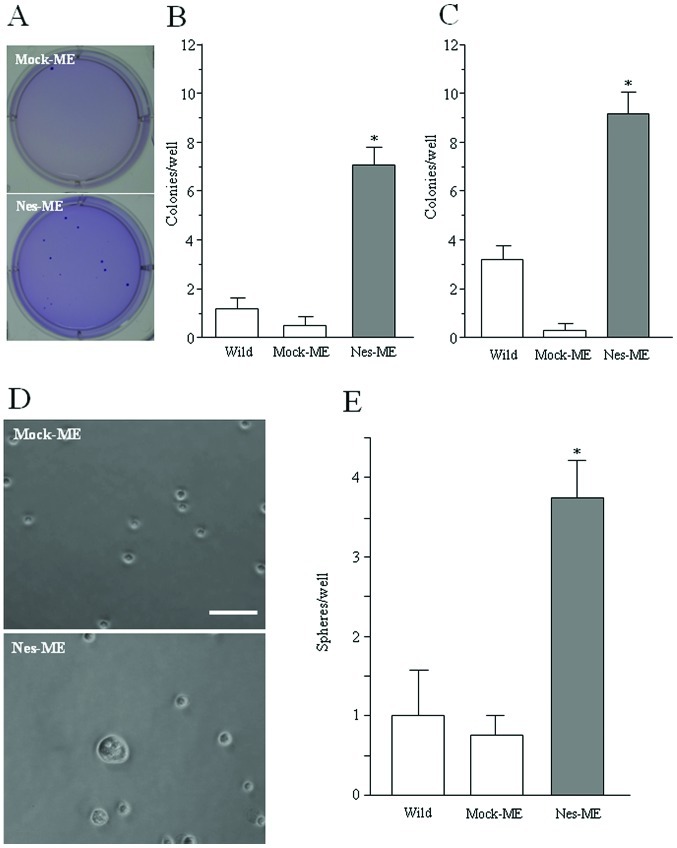
Anchorage-independent cell proliferation and sphere formation assays of nestin-transfected ME-180 cells. (A–C) In the soft agar assay, Nes-ME cells formed more colonies than Mock-ME and wild cells. (D) Nestin-transfected ME-180 cells formed larger and (E) more numerous spheres than mock cells. (D) Phase-contrast images: bar, 100 *μ*m; ^*^P<0.0001.

To examine the effect of increased expression of nestin in ME-180 cells on colony formation, a soft agar assay was performed. Nes-ME cells cultured with 1×10^3^ (Fig. 7B) and 2×10^3^ cells/well (Fig. 7A and C) formed more colonies than Mock-ME and wild cells in 60 days (P<0.0001).

### Sphere forming assay

To determine whether cervical cancer cells had characteristics of CSCs, a sphere formation assay was performed. Cells were plated in ultra-low attachment plates supplemented with EGF and bFGF, and then cultured for 7 days. Nes-ME cells formed larger size (Fig. 7D) and more numerous spheres than Mock-ME cells (P<0.0001) (Fig. 7E).

## Discussion

In this study, nestin was absent or only faintly expressed in normal squamous epithelial cells. Nestin expression was originally identified in neuroepithelial stem cells and neural cells (5). In pathological conditions, nestin is expressed in repair processes in the CNS, muscle, liver (44–47) and infarcted myocardium (48). In adult organisms, nestin-expressing cells are restricted to defined locations, where they may function as a cellular ‘reserve’ capable of proliferation, differentiation and migration after reactivation (49,50). We found that nestin was slightly to moderately expressed in cervical hyperplasia and squamous metaplasia lesions of chronic cervicitis (data not shown). These findings suggest that nestin does not affect squamous cell functions in normal cervical tissues, but there is a possibility that nestin plays a role in reserve cells active in cervical inflammation.

Nestin expression levels correlated with CIN stage. A significant positive correlation exists between VEGF-A, VEGF-C, VEGF-D and VEGFR-3 expression and stage of cervical carcinogenesis (51). CIN 2,3 exhibited higher VEGF mRNA, transforming growth factor (TGF)-β1 and TGF-β receptor 1 levels than CIN 1 (52). Expression of insulin-like growth factor 1 receptor (IGF-1R) and phosphorylated IGFR-1 positively correlated with CIN grade (53). Our group reported that fibroblast growth factor receptor 2 IIIc (FGFR2 IIIc) expression was increased in advanced CIN stage (4). In the current study, the nestin expression pattern correlated with CIN stage, similar to the above-mentioned growth factors and receptors. Concerning precancerous lesions, activation of oncogenic K-ras in the nestin cell lineage in pancreatic tissue is sufficient for initiation of premalignant pancreatic intraepithelial neoplasia (PanIN) lesions in mice. These findings may indicate that nestin expression is closely related to the formation of precancerous lesions including the growth of neoplastic cells in CIN and carcinogenesis. Further research is needed to clarify the roles of nestin in precancerous lesions and carcinogenesis.

Previously, we found nestin immunoreactivity within cancer cells in ∼30% of pancreatic ductal adenocarcinoma cases (6). In prostate cancer, nestin is expressed in 75% of lethal androgen-independent prostate cancer cases (27). Among breast cancer subtypes, nestin is highly expressed in the basal subtype (ERα^−^/PR^−^/Her2^−^), but not in the Her2 subtype (ERα^−^/PR^−^/Her2^+^) or luminal epithelial subtype (ERα^+^/PR^+^) (26). In contrast to these malignant tumors, nestin was expressed in all cervical cancer cases, unrelated to their histological type. Although the etiology of nestin expression pattern in cervical cancer is unclear, we suggest that nestin may play a fundamental and important role in cervical carcinogenesis.

The CSC hypothesis suggests that only CSCs within the tumor can self-renew and proliferate extensively to form new tumors (54) and that CSCs are considered to be an attractive target for advanced cancer therapy (55). In cervical cancer, as in other cancers, characterization of CSCs will allow for the development of new treatments that are specifically targeted against this critical population of cells, particularly their ability to self-renew, resulting in more effective therapies (54). Previous studies suggested that CD44^+^CK17^+^/sphere-forming cervical cancer cells display stem cell properties (54). Using a sphere culture method that favors the growth of self-renewing cells, sphere-forming cells of cervical cancer showed an expression pattern of CD44^high^/CD24^low^ that resembles the CSC surface biomarker of breast cancer (55). Non-adherent spheres of cervical cancer maintained or increased ALDH and stem cell markers such as Nanog, nestin and Oct4 (38). Cells with self-renewal capability are able to sustain growth in suspension giving rise to non-adherent colonies (38). These results suggest that expression levels of nestin in cervical cancer cells closely correlate with CSC function.

In summary, nestin expression correlates with CIN progression and was expressed in all cervical cancer specimens examined. Furthermore, nestin stimulated colony and sphere formation *in vitro*. These findings suggest that nestin plays important roles in carcinogenesis and tumor formation of cervical cancer cells through regulation of CSC functions.

## Figures and Tables

**Table I:** Clinical features of cervical cancer patients.

Age (years)	29–72
Median	50
Mean	50.4
FIGO stage^a^	
Ia	4
Ib	30
IIa	3
IIb	14
IIIa	1
IIIb	3
Keratinization	
Absent	34
Present	21
Nodal metastasis	
Absent	37
Present	18
HPV infection	
Absent	3
Present	23
Not examined	29

a FIGO, International Federation of Gynecology and Obstetrics.
